# Bonding of Resin Cement to Zirconia with High Pressure Primer Coating

**DOI:** 10.1371/journal.pone.0101174

**Published:** 2014-07-03

**Authors:** Chen Wang, Li-na Niu, Ying-jie Wang, Kai Jiao, Yan Liu, Wei Zhou, Li-juan Shen, Ming Fang, Meng Li, Xiang Zhang, Franklin R. Tay, Ji-hua Chen

**Affiliations:** 1 State Key Laboratory of Military Stomatology, Department of Prosthodontics, School of Stomatology, Fourth Military Medical University, Xi'an, P. R. China; 2 State Key Laboratory of Military Stomatology, Department of General and Emergency, School of Stomatology, Fourth Military Medical University, Xi'an, P. R. China; 3 State Key Laboratory of Military Stomatology, Department of Oral Anatomy and Physiology, School of Stomatology, Fourth Military Medical University, Xi'an, P. R. China; 4 Department of Endodontics, College of Dental Medicine, Georgia Regents University, Augusta, Georgia, United States of America; UNC School of Dentistry, University of North Carolina-Chapel Hill, United States of America

## Abstract

**Objectives:**

To investigate the effect of air-drying pressure during ceramic primer coating on zirconia/resin bonding and the surface characteristics of the primed zirconia.

**Methods:**

Two ceramic primers (Clearfil Ceramic Primer, CCP, Kuraray Medical Inc. and Z-Prime Plus, ZPP, Bisco Inc.) were applied on the surface of air-abraded zirconia (Katana zirconia, Noritake) and dried at 4 different air pressures (0.1–0.4 MPa). The primed zirconia ceramic specimens were bonded with a resin-based luting agent (SA Luting Cement, Kuraray). Micro-shear bond strengths of the bonded specimens were tested after 3 days of water storage or 5,000× thermocycling (n = 12). Failure modes of the fractured specimens were examined with scanning electron miscopy. The effects of air pressure on the thickness of the primer layers and the surface roughness (S_a_) of primed zirconia were evaluated using spectroscopic ellipsometry (n = 6), optical profilometry and environmental scanning electron microscopy (ESEM) (n = 6), respectively.

**Results:**

Clearfil Ceramic Primer air-dried at 0.3 and 0.4 MPa, yielding significantly higher µSBS than gentle air-drying subgroups (p<0.05). Compared to vigorous drying conditions, Z-Prime Plus air-dried at 0.2 MPa exhibited significantly higher µSBS (p<0.05). Increasing air-drying pressure reduced the film thickness for both primers. Profilometry measurements and ESEM showed rougher surfaces in the high pressure subgroups of CCP and intermediate pressure subgroup of ZPP.

**Conclusion:**

Air-drying pressure influences resin/zirconia bond strength and durability significantly. Higher air-drying pressure (0.3-0.4 MPa) for CCP and intermediate pressure (0.2 MPa) for ZPP are recommended to produce strong, durable bonds between resin cement and zirconia ceramics.

## Introduction

Interest for using advanced ceramic materials for dental applications including orthodontic brackets, posts, implant abutments, and restoration frameworks has increased substantially in recent years. Among the different types of ceramic materials, zirconia has attracted the most attention because of its superior mechanical properties and esthetics [1]–[5]. Chemical inertness is one of the advantages of zirconia, which enables it to survive the harsh chemical change in oral environments. However, it is also the chemical inertness which poses a big challenge for the bonding of zirconia. Unlike silica-based ceramics, the surface stability of zirconia makes it difficult to be chemically bonded to resin cements [4].

Due to the lack of amorphous silica in zirconia, traditional bond strength-increasing methods, such as the application of hydrofluoric acid to selectively dissolve the silica phase, and the use of silane coupling agent to create chemical bonding between the silica phase and resin cements, are not suitable for the bonding of zirconia [6]. Thus, new methods focused on surface modification and activation of the stable chemical surface of zirconia have been investigated to create chemical reaction at the cement/zirconia interface. These methods include chemical vapor deposition, selective infiltration etching, laser irradiation, fluorination and “glaze on” technique [6]–[10]. One promising method is the use of tribochemical silica-coating system to introduce silica phase onto the zirconia surface [4], [7]. Although increases in immediate bond strength between zirconia and composite resin was achieved with this method [11]–[13], this surface pre-treatment method showed significant loss in long-term bond strength when compared with the combined application of airborne-particle abrasion and resin luting agents containing phosphate ester monomers (10-methacryloyloxydecyl dihydrogen phosphate, 10-MDP) on the zirconia surface [1]–[3], [7], [14]–[18]. For the latter method, it is thought that the hydroxyl groups present on the zirconia surface could react with the phosphate group of 10-MDP. This results in the formation of a stable Z-O-P chemical bond between zirconia ceramics and 10-MDP [19]; the zirconia surface become an organic-inorganic hybrid composite [20] which can react with the polymerizable groups of methacrylate resin composites, producing higher bond strength and better durability. In addition, it was shown that particle air-abrasion or tribochemical coating, followed by the application of 10-MDP-containing bonding agents, resulted in increased bond strength compared to 10-MDP-containing cements only. The large reaction surface, high concentration of functional terminal groups, and intimate contact between the two reactants all contribute to the formation of Zr-O-P bonds and the improved zirconia-resin bonding [20].

Recently, 10-MDP was introduced into ceramic primers and used together with carboxylic acids, silanes or other adhesive resin monomers to promote chemical bonding to zirconia [21]. It is believed that inclusion of all the reagents in a single bottle plays distinctive roles in zirconia-resin bonding, with 10-MDP used for strong chemical bond formation, silane for surface energy modification and wetting [22] and HEMA as a co-solvent [23]. To avoid phase separation between hydrophilic and hydrophobic methacrylate resin components, manufacturers also add volatile solvents such as ethanol when formulating zirconia primers. Although improved bond strength was reported with one-bottle zirconia primers [24], excess residual solvent on the primer-treated zirconia surface inhibits polymerization of resin monomers and interferes with the durability of resin-zirconia bonding [25]. Since decreasing the solvent content in a zirconia primer may cause phase separation and compromise its wetting properties, modification of the primer-coating protocol appears to be feasible in alleviating this problem. Although gentle air-drying was recommended by manufacturers to evaporate solvent during primer-coating, recently studies showed that solvent removal was better achieved via more aggressive treatments such as warm air-drying or hot water-rinse. These strategies resulted in thinner and more uniform primer films which are critical for resin-zirconia bond durability [26]–[30]. To date, there is no consensus on the most appropriate air-drying pressure that evaporates zirconia primer solvents without adversely affecting bond stability. Thus, the present study investigated the effects of air-drying pressures in zirconia primer-coating protocols on the physicochemical properties of two commercial ceramic primers and the bond strength of the cement/zirconia interface. The null hypothesis tested was that air-drying pressure has no effect on the strength of zirconia-resin bonds and the surface characteristics of the primed zirconia.

## Materials and Methods

### Specimen preparation

#### Zirconia surface treatment

Zirconia cylinders (Katana zirconia, Noritake Dental Supply Co. Ltd., Miyoshi, Japan) were cut into disk-like specimens (5 mm diameter and 2 mm thick) and embedded in epoxy resin blocks (Epon 812; Fluka, Buchs, Switzerland). The surface of the zirconia specimens were polished with silicon carbide papers (from 180-grit to 2000-grit) under water cooling, air-abraded with 110 µm Al_2_O_3_ particles (Saint-Gobain Ceramic Materials Corp., Osaka, Japan.) under a pressure of 0.3 MPa (Combilabor, CL-FSG94, Heraeus Kulzer, GmbH & Co. KG, Hanau, Germany) for 15 s with the tip inclined approximately 45° and at a distance of 10 mm from the zirconia surface, and ultrasonically cleaned in 99% ethanol for 10 min [31], [32].

#### Primer coating

One hundred and ninety-two specimens were randomly divided into two groups according to the primer used: a silane/10-MDP-combined primer (Clearfil Ceramic Primer; CCP; Kuraray Medical Inc., Tokyo, Japan) and a primer consisting of 10-MDP and carboxylic acid monomers (Z-Prime Plus; ZPP, Bisco Inc., Schaumburg, IL). Each group was divided into four subgroups according to the air-dried pressures used (0.1, 0.2, 0.3 or 0.4 MPa). One drop of the ceramic primer was applied on the surface of the specimen, left to react for 1 min and air-dried for 10 s with the nozzle swept and inclined approximately 45° and at a distance of 10 mm from the surface. A pressure gauge was connected to the triple syringe to control the pressure of the output oil-free air.

#### Bonding procedures

To control the bonding area for micro-shear bond test, tygon tubes (R-3603, Norton Performance Plastic, Cleveland, OH, USA) with an internal diameter of 0.8 mm and 2 mm in height were placed on the primed zirconia surfaces. The lumens were then carefully filled with a 10-MDP containing resin cement (Clearfil SA Luting, Kuraray Medical Inc.). The resin cement was light-cured for 40 s using a light-curing unit (Translux CL, Heraeus Kulzer, Dormagen, Germany) with an output intensity of 800 mW/cm^2^. All specimens were stored in distilled water for 3 days at 37°C. Half of the specimens in each group were used for immediate micro-shear bond strength test and the other half of the specimens were subjected to thermocycling aging treatment before bond testing (n = 12). Thermocycling was performed for 5,000 cycles between 5.0±0.5°C and 55±0.5°C, with 20 s dwell time in each water bath, and 2 s in transfer time [31]. The main materials utilized and their characteristics are listed in Table S1 and the groups and primer coating protocols used in the present study are presented in Table S2.

### Micro-shear bond strength test (µSBS)

A universal testing machine (Model AGS-10KNG, Shimadzu, Tokyo, Japan) equipped with a customized fixture for stabilizing the specimen and for gripping the resin cement cylinder was used for micro-shear bond strength test (Figure S1). Loading was applied with a cross-head speed of 0.5 mm/min until failure occurred. The maximum force (N) to produce fracture was recorded and the mean stress values on failure (in MPa) were calculated for all subgroups (Table 1).

**Table 1:** Means and standard deviation of µSBS for various primers under different air-drying pressures and storage conditions (n = 12).

Primer	Air-drying pressure (MPa)	After 3 days of water storage	After 5,000× thermocycling
Clearfil Ceramic Primer	0.1	18.7 (6.3)^A^_a_	12.1 (5.1)^AB^_b_
	0.2	19.7 (3.9)^A^_a_	11.4 (1.9)^AB^_b_
	0.3	35.7 (7.0)^B^_a_	32.4 (6.5)^D^_a_
	0.4	28.4 (4.4)^B^_a_	23.5 (5.8)^C^_a_
Z-Prime Plus	0.1	20.5 (8.6)^A^_a_	15.8 (2.9)^B^_b_
	0.2	29.6 (5.2)^B^_a_	21.6 (2.7)^C^_b_
	0.3	20.3 (3.1)^A^_a_	14.9 (4.0)^B^_b_
	0.4	13.7 (5.9)^A^_a_	8.7 (3.9)^A^_b_

Note: Within the same column, groups with the same upper case superscript letters are not statistically different (p>0.05). Within the same row, groups with the same lower case subscript letters are not statistically different (p>0.05).

The fracture surface of representative specimens was sputter-coated with gold–palladium (E-1030, Hitachi, Tokyo, Japan) and examined using scanning electron microscopy (SEM, S4800, Hitachi, Japan). Failure modes were recorded as: adhesive failure (failure occurring at the zirconia-cement interface) and cohesive failure (failure within cement). Failure areas of each mode were calculated as described previously [16] and expressed as a percentage of the total bonding surface area for each subgroup.

### Primer film characterization

#### Thickness measurement

The effect of air-drying pressure on the thickness of the coated primer film was determined using a spectroscopic ellipsometer (PZ2000, Jobin Yvon S.A.S, France) equipped with a He-Ne laser source (λ = 632.8 nm) at 70° incidence angle [26]. Before testing, the primer was applied onto smooth (R_a_<10 nm) and highly reflecting electropolished silicon wafer (10 mm×10 mm), and then air-dried with different pressures (0.1, 0.2, 0.3 or 0.4 MPa; n = 6) for 10 s. Specimens which were only applied with primer but without air-drying procedures were used as the control.

#### Surface roughness measurement

The effect of air-drying pressure on the average surface roughness (S_a_) of the primer-coated zirconia was examined with an optical profilometer (Talysurf CCI 6000, Taylor Hobson, Paoli, PA). Briefly, the primer was applied on the air-abraded zirconia, left to react for 1 min and air-dried for 10 s with different pressures (0.1, 0.2, 0.3 or 0.4 MPa; n = 6). Air-abraded zirconia specimens without primer application and air-drying were used as the control. Three-dimensional images in 3 different regions (350 µm×350 µm) of each zirconia surface were obtained with the optical profilometer and the surface roughness was analysed with the TalyMap Gold software (Taylor Hobson).

#### Environmental scanning electron microscopy

The specimens used in section 2.3.2 were further analysed with a scanning electron microscope (ESEM, Quanta 600 FEG, FEI, The Netherlands) at 10 kV in low vacuum mode (4.6 Torr) to characterize the topographical changes of zirconia surfaces.

### Statistical analysis

Data from the µSBS test were analysed with three-way analysis of variance (ANOVA) to examine the effects of primer type, air-drying pressure, thermocycling, and the interaction of these three factors on bond strength. The two-way ANOVA was employed for analysing the data (effects of primer type and air-drying pressure) derived from the primer film thickness measurement and surface roughness measurement. For these analyses, post-hoc pairwise comparisons were performed using the Tukey test. The Pearson correlation test was performed to evaluate the significance of the correlation among the primer film thickness, surface roughness and air-drying pressure. Statistical analyses were performed using the SPSS 13.0 software package (SPSS 13.0 for Windows, SPSS Inc., Chicago, IL). All for analyses, statistical significance was preset at α = 0.05.

## Results

### Micro-shear bond strength

The results of µSBS tests are shown in Table 1. The main effects of the primer type, air-drying pressure and thermocycling, and the interaction of any two of these three factors on the µSBS values were all significant (p<0.01). The interaction of all the three factors on µSBS values was not significant (p>0.05). Tukey's multiple comparisons indicated that at 0.1 MPa (gentle) pressure, there were no statistically significant differences in the immediate µSBS between Clearfil Ceramic Primer (CCP) group and Z-Prime Plus (ZPP) group (p>0.05). As the air-drying pressure increased to 0.3 and 0.4 MPa (high), the immediate µSBS of the CCP subgroups were significantly improved compared to those at 0.1 MPa (gentle) and 0.2 MPa (intermediate) pressure (p<0.05). For the ZPP, the immediate µSBS achieved the highest value at 0.2 MPa air pressure (p<0.05) and then declined when the air-drying pressure was further increased to 0.3 and 0.4 MPa (p<0.05).

After thermocycling, for the CCP group, decrease in µSBS after thermocycling only occurred in the 0.1 MPa and 0.2 MPa subgroups (p<0.05), while for the 0.3 MPa and 0.4 MPa subgroups, there were no significant differences between the µSBS before and after thermocycling (p>0.05). The highest µSBS in the CCP group after thermocycling was achieved in the 0.3 MPa subgroups (32.4 MPa) (p<0.05). For the ZPP group, the µSBS of different air-pressure subgroups were all significantly decreased after thermocycling (p<0.05), with the 0.4 MPa subgroup being the lowest (8.7±3.9 MPa) and the 0.2 MPa subgroup being the highest (21.6±2.7 MPa) (p<0.05).

### Failure mode analysis

**Figure 1:**
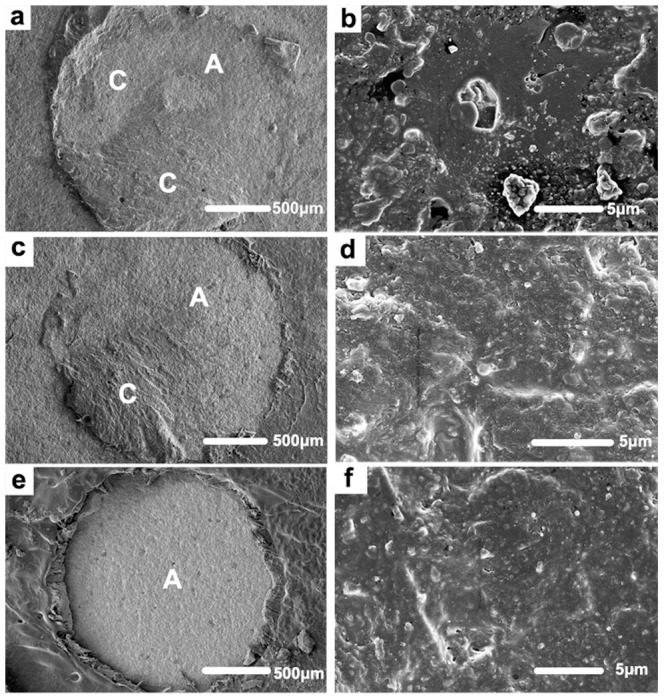
Scanning electron microscopy images of representative failure modes in the CCP and ZPP subgroups. (**a, b**) low and high magnification images of a mixed failure mode in the CCP-0.3 subgroup after thermocycling, with a large percentage of cohesive failure at the bonding interface and small area of primer remnant on the zirconia surface; (**c, d**) low and high magnification images of a mixed failure mode in the ZPP-0.1 subgroup without thermocycling, with a small percentage of cohesive failure mode; (**e, f**) low and high magnification images of complete adhesive failure in CCP-0.1 subgroup after thermocycling, with primer residues on the zirconia surface. (**a, c, e**) 50× magnification; (**b, d, f**) 5,000× magnification. **A**: adhesive failure from zirconia surface; **C**: cohesive failure in SA Luting resin cement.

**Figure 2:**
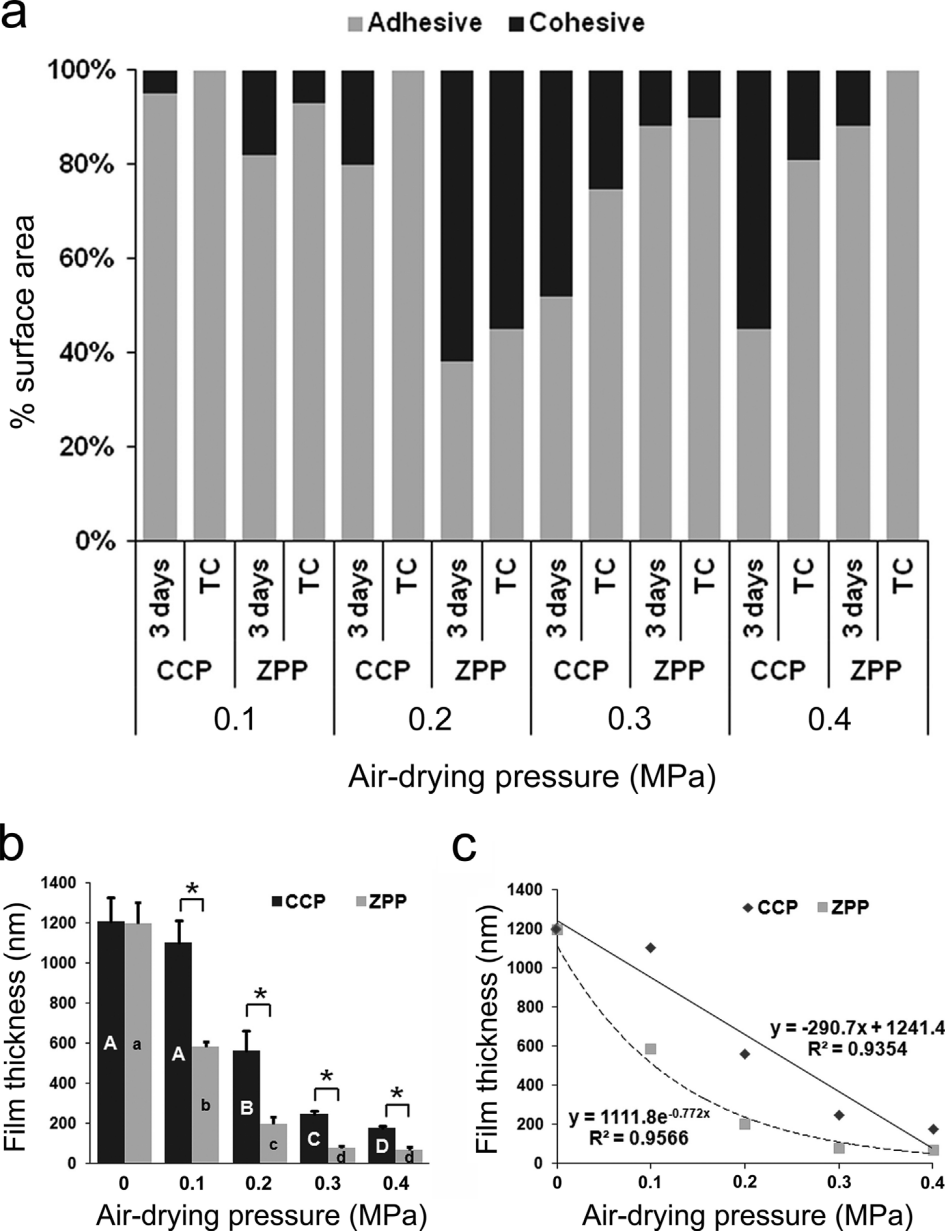
Failure modes analysis and primer film thickness result. (**a**) Failure modes for the micro-shear bonding test after 3 days of water storage or 5,000 thermocycles. (**b**) Variation in primer film thickness as a function of air-drying pressure. Data presented are means and standard deviations (n = 6). For CCP subgroups, the same upper case letters represent absence of statistically significant difference (p>0.05). For ZPP subgroups, the same lower case letters represent absence of s statistically significant difference (p>0.05). Asterisks indicate significant differences between the two ceramic primers (p<0.05). (**c**) Correlations between primer film thickness and air-drying pressure for the CPP and ZPP ceramic primers.

Scanning electron microscopy images of representative failure modes are shown in Figure 1 and mean percentages of areas assigned to the failure modes observed in the subgroups are shown in Figure 2a. For the immediate testing groups (i.e. after 3-day water storage), only the ZPP-0.2 MPa, CCP-0.3 MPa and CCP-0.4 MPa subgroups showed high percentage of cohesive failure on the zirconia surface. After 5,000 thermocycles, the percentage of adhesive failure increased in all subgroups. Only the ZPP-0.2 MPa subgroups indicated high percentages of cohesive failure, while the CCP-0.1 MPa, CCP-0.2 MPa and ZPP-0.4 MPa subgroups showed completely adhesive failure. Figures 1a and 1b represent low and high magnification images of a mixed failure mode in the CCP-0.3 MPa subgroup after thermocycling, with a large percentage of cohesive failure at the bonding interface and small area of primer remnant on the zirconia surface; Figures 1c and 1d represent low and high magnification images of a mixed failure mode in the ZPP-0.1 MPa subgroup without thermocycling, with a small percentage of cohesive failure mode. Figures 1e and 1f represent low and high magnification images of complete adhesive failure in CCP-0.1 MPa subgroup after thermocycling, with primer residues on the zirconia surface.

### Primer film thickness

The results of primer film thickness (Figure 2b) revealed that for both primers, increase in air-drying pressure gave rise to a significant thickness reduction (p<0.05). Subgroups treated with ZPP showed significantly thinner primer films than those treated with CCP at different air-drying pressures (p<0.05). For CCP, primer film thickness decreased from 1103 nm in the 0.1 MPa subgroup to 178 nm in the 0.4 MPa subgroup. For ZPP, primer film thickness decreased from 584 nm in the 0.1 MPa subgroup to 69 nm in the 0.4 MPa subgroup. A significant linear correlation was found between air-drying pressure and primer film thickness for CCP (r = −0.967, p = 0.047), while a significant power correlation was identified between air-drying pressure and primer film thickness for ZPP (r = −0.911, p = 0.031) (Figure 2c).

### Surface roughness measurement

**Figure 3:**
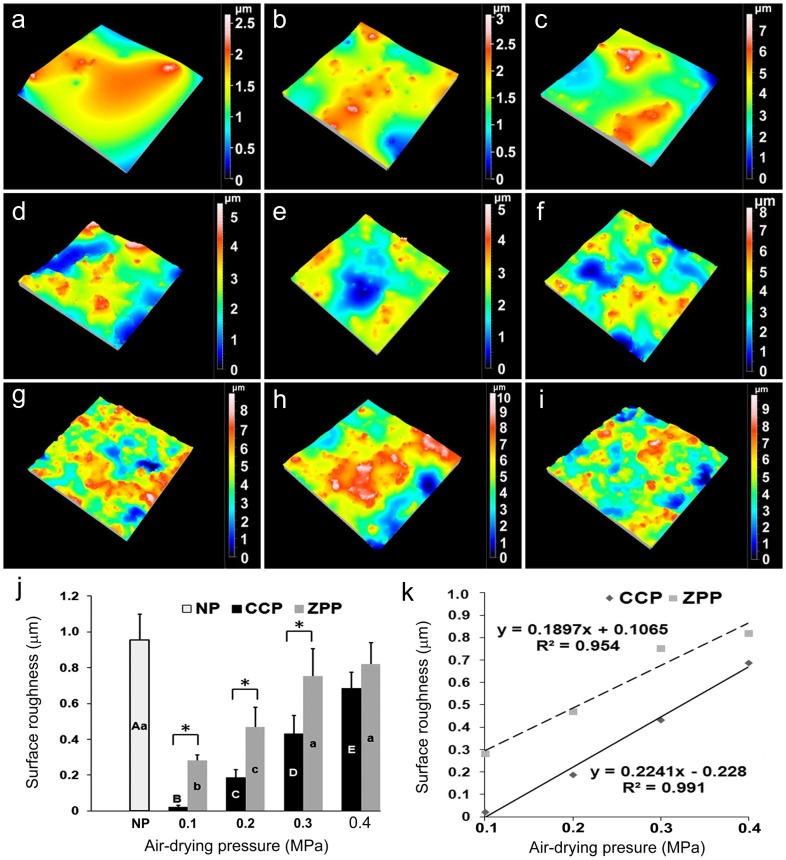
Surface roughness of specimens in subgroups. (**a–h**) 3D surface images (350 µm×350 µm) of specimens obtained from optical profilometry in subgroups: (a) CCP-0.1 MPa; (b) CCP-0.2 MPa; (c) CCP-0.3 MPa; (d) CCP-0.4 MPa; (e) ZPP-0.1 MPa; (f) ZPP-0.2 MPa; (g) ZPP-0.3 MPa; (h) ZPP-0.4 MPa; (**i**) NP. (air blasted zirconia without primer application) (**j**) Mean roughness (S_a_) of experimental subgroups and control NP in µm. Data are presented as means and standard deviations (n = 6). For CCP subgroups, the same upper case letters represent absence of statistically significant difference (p>0.05). For ZPP subgroups, the same lower case letters represent absence of s statistically significant difference (p>0.05). Asterisks indicate significant differences between the two ceramic primers (p<0.05). (**k**) Correlations between surface roughness and air-drying pressure in the CPP and ZPP ceramic primers.

Figures 3a–3i are representative 3D roughness profiles after primer coating in various subgroups. The mean roughness (S_a_) values of the experimental subgroups and sandblasted zirconia without primer application (NP) are shown in Figure 3j. The mean roughness values of the CCP subgroups were significantly lower than those of the ZPP subgroups. For the CCP subgroups, surface roughness of the sandblasted zirconia was significantly decreased after primer coating, with the CCP-0.1 MPa subgroup exhibiting the lowest surface roughness (p<0.05). For the ZPP subgroups, surface roughness of the sandblasted zirconia was significantly decreased in the ZPP-0.1 MPa and ZPP-0.2 MPa subgroups (p<0.05). Significant linear correlations were observed between air-drying pressure and surface roughness in the CCP subgroups (r = 0.996, p = 0.04) and the ZPP subgroups (r = 0.977, p = 0.023) (Figure 3k).

### Environmental scanning electron microscopy

**Figure 4:**
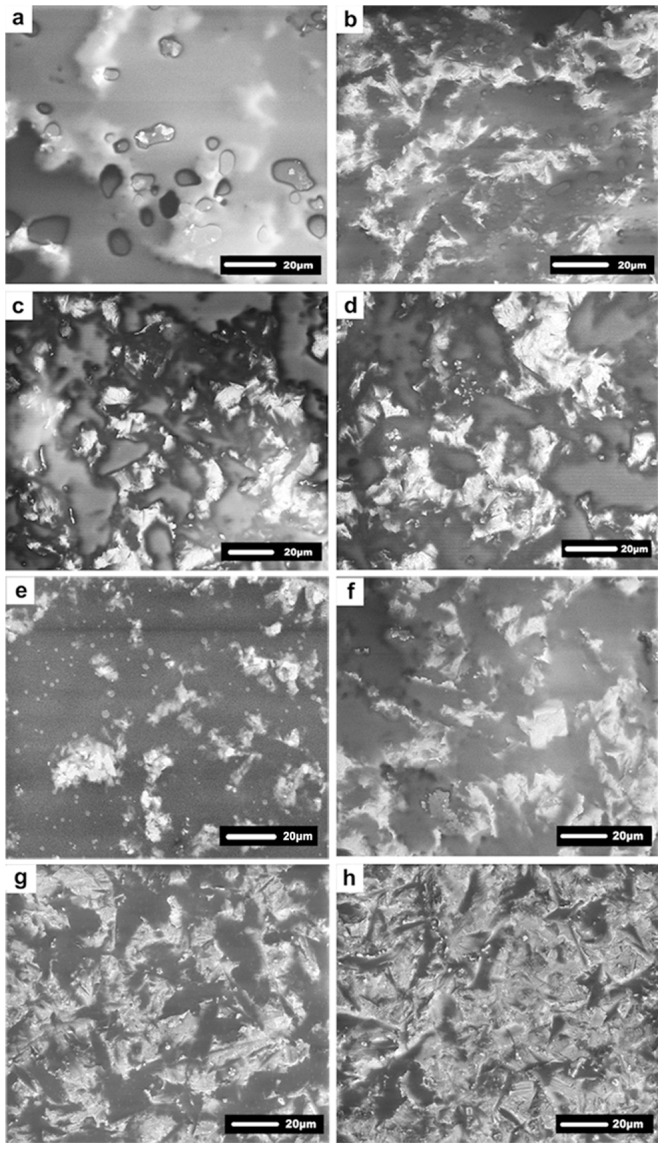
Representative environmental scanning electron microscopy of primed, air-blasted zirconia surfaces in subgroups. (a) CCP-0.1 MPa; (b) CCP-0.2 MPa; (c) CCP-0.3 MPa; (d) CCP-0.4 MPa; (e) ZPP-0.1 MPa; (f) ZPP-0.2 MPa; (g) ZPP-0.3 MPa; (h) ZPP-0.4 MPa.

The results of ESEM observation (Figure 4) of the surface topography of primer-coated zirconia were consistent with data derived from surface roughness measurements. For the CPP subgroups, the CCP-0.1 MPa subgroup showed a thick layer of primer containing some bubbles (Figure 4a). With increases in air-drying pressure, primer films were thinned more evenly and the irregular contour of the sandblasted zirconia surface was visible gradually (Figures 4b–4d). Compared with the CCP subgroups, primer films in the ZPP subgroups (Figures 4e–4h) were much thinner, resulting in rougher primed zirconia surfaces. As the air-drying pressure increased, the primer-coated zirconia surface became increasingly rougher.

The raw data of micro-shear bond strength, primer film thickness and surface roughness are shown in Data S1.

## Discussion

The present *in vitro* study evaluated the effect of air-drying pressures during primer coating on the resin-zirconia bond strength and the surface characteristics of the primed zirconia surfaces. It was found that higher air-drying pressures during primer coating resulted in thinner primer layers, rougher surfaces of the primed zirconia, higher bond strength and better durability of the bond between resin cement and primed zirconia. Thus, the null hypothesis that air-drying pressure has no effect on the bond strength of the zirconia/resin bonding and the surface characteristics of the primed zirconia has to be rejected.

110 µm Alumina powder sandblasting was performed to standardize the zirconia surface in the present study. Although the negative effect of the microcracks on the zirconia surface caused by this surface pretreatment, sandblasting is believed beneficial to zirconia bonding by increasing the surface roughness, bonding area and remove any contaminants from the ceramic surface [1], [4], [7]. MDP-containing resin cements are also recommended for luting bonding of zirconia for clinical use [14]. However, according to previous laboratory investigations, the shear bond strengths for MDP-containing cements to sandblasted zirconia without primer coating were lower than the assumed minimal bond strength for acceptable clinical bonding to ensure favourable clinical service [21], [24], [33]. In the present study, chemical modification of the sandblasted zirconia surface, a combination of both surface pretreatments, is performed to try achieving higher bond strength [4], [7]. The chemical bond strategies involve the use of acid adhesive monomers presented in adhesive cementation systems including a primer (such as MDP, 4-META, MEPS, and zirconate coupler) [4], [7], [15], [21]. Clearfil Ceramic Primer (CCP) used in the experiment is a two component ceramic primer, combining 3-methacryloxypropyltrimethoxysilane (3-MPS) and 10-MDP dissolved in ethanol. The 10-MDP resin monomers in CCP form cross-linkages with methacrylate functional groups in the SA Luting cement as well as with the OH-groups on the ceramic surface and silanol [1], [7], [14], [34]. The 10-MDP monomers also create an acid environment that may promote rapid 3-MPS condensation, enhance zirconia surface wettability and resist moisture sorption [35]. Thus, one would have anticipated that the use of CCP on air-abraded zirconia produced better bonding performance. Contrary to this expectation, poor bonding strength and low durability were achieved in previous investigations when gentle priming protocols were employed to apply CCP on air-abraded zirconia surface [24], [36]. In the present study, it was found that both the immediate µSBS and the durability of the resin-to-zirconia bonding in the CPP group were significantly increased when the ceramic primer was air-dried with higher pressure (0.3 or 0.4 MPa). This phenomenon may be attributed to the formation of a thinner primer layer under higher air-drying pressure. As air-drying pressure increases, the thickness of the CCP layer decreases and the surface roughness of the primed zirconia increases significantly. This may result in better micromechanical interlocking of resin and higher bond strength. In addition, the silane component in CCP, which is not chemically bonded to the zirconia, may be hydrolysed easily if it is present abundantly on the external surface. The free silane may be eliminated by high pressure air-drying, leaving a monolayer of strong oligomeric siloxane in the innermost region of the bonded interface that is more resistant to hydrothermal attack [26]. When CCP is air-dried with gentle pressure, a thick primer layer is formed the completely covers the air-abraded zirconia surface. This may result in decreases in surface roughness and compromised microretention of the resin cement, thereby compromising bond strength. Because a thick primer film is less incapable of resisting hydrolytic degradation and absorbing mechanical stress without causing crack growth into the primer film [26], [27], deterioration of bond durability occurs. Apart from primer thickness, more thorough elimination of residual solvents from the primer layer in the presence of stronger air-drying pressures may also contribute to the bonding effectiveness of the CCP group. Removal of water, ethanol and other by-products that remain in the pores of the silane network may also increase the number of bonding sites available for reacting with 10-MDP and enhance Zr-O-P bond formation [28].

Z-Prime Plus is a proprietary formula of both nonvolatile hydrophilic (MDP, carboxylic monomer and HEMA) and hydrophobic (Bis-GMA) resin monomers dissolved in ethanol and water. Carboxylic monomer is another acid adhesive resin monomer employed in ZPP to assist 10-MDP in chemically interacting with zirconia [8], [24]. Previous studies have shown that this primer yields superior bond strength when employed for zirconia-resin bonding [24], [37]. However, in the present study, when Z-Prime Plus was air-dried with gentle pressure, there was no significant difference between the µSBS of the ZPP group and CCP group. This may be due to the compositional mismatch between Z-Prime Plus (Bisco Inc.) and the SA Luting resin cement (Kuraray Medical Inc.) [24]. Similar to the CCP group, the thickness of the ZPP layer decreased and the surface roughness of the primed zirconia increased, as the air-drying pressure increased. However, the highest µSBS between ZPP-primed zirconia and the resin cement was achieved when the primer was air-dried with intermediate pressure (0.2 MPa), but not with stronger air- pressure (0.3-0.4 MPa). The different responses of the two primers to air-drying pressure may be attributed to the differences between the two materials in terms of monomer composition, initiator and solvent [24], [38]. Unlike CCP, ZPP does not contain silane and has a lower viscosity. Thus, compared with the more viscous CCP, it is much easier for ZPP to be thinned to a monolayer and to eliminate the residue solvent using a lower pressure. Thus, it appears that 0.2 MPa is the optimal air-drying pressure for ZPP that is able to form strong bonds between resin cement and zirconia ceramics. Further increasing the air-drying pressure may result in a very thin or virtually invisible nonexistent primer film that exposes the air-abraded zirconia surface without efficient priming, thereby decreasing bond strength.

After thermocycling, a significant reduction in bond strength and predominant adhesive failure were found in all subgroups of ZPP. Hydroxyethyl methacrylate, the co-solvent present in ZPP, is included in the ceramic primer for minimizing phase separation and increasing the miscibility of hydrophobic and hydrophilic resin components [29], [39]. However, because of the existence of HEMA, the primer may be more susceptible to hydrolytic degradation [40] and disruption of the cross-linkages between ZPP and zirconia after thermocycling. It is known that HEMA is a non-volatile resin monomer that is difficult to be removed by air-drying [29], [41]. Further studies are required to develop primer coating protocols to promote the hydrolytic stability hydrolytically of HEMA-containing ceramic primers.

It must be noted that only one aging condition (thermocycling) was performed to evaluate the durability, stability, and hydrolytic degradation of the resin-ceramic bond. This may be considered one of the limitations of the current study as long-term water storage may significantly affect the bonding durability [14], [36], [42]. Further investigation should consider a combination of long-term water storage and thermal cycling at regular intervals to test the long-term durability of the bonding because none of these artificial ageing treatments alone can realistically simulate the clinical scenario.

## Conclusions

Within the limitations of the present study, it may be concluded that application of a relative high air-drying pressure during ceramic priming is necessary to achieve strong and long-term bonding between resin cement and zirconia. There exists an optimal air-drying pressure for each ceramic primer for forming strong durable bonds between resin cement and zirconia ceramics.

## Supporting Information

Figure S1 Schematic diagram of micro-shear test. (**a**) Prior to micro-shear bond test, the tygon tubes around the resin cement cylinders were removed, by gently cutting the tubes into two hemi-cylinders using a feather blade. (**b**) Specimens were clamped with the custom fixture and tested in a universal testing machine (EZ-Test 500 N, Shimadzu, Kyoto, Japan). A thin wire (diameter 0.2 mm) was looped around the resin cement cylinder, making contact through half of its circumference and was gently held flush against the resin/zirconia interface. A shear force was applied to each specimen at a cross-head speed of 0.5 mm/min until failure occurred. The resin-zirconia interface, the wire loop and the center of the load cell were aligned as straight as possible to ensure the desired orientation of the applied shear test force.
(TIF)

Table S1 List of materials used in the present study.
(DOC)

Table S2 Subgroups and primer-coating protocols used in the present study.
(DOC)

Data S1 The raw data of micro-shear bond strength, primer film thickness and surface roughness in this study.
(XLS)

## References

[1] Kern M (2009) Resin bonding to oxide ceramics for dental restorations. J Adhes Sci Technol 23: 1097–1111.

[2] Abou Tara M, Eschbach S, Wolfart S, Kern M (2011) Zirconia ceramic inlay-retained fixed dental prostheses-first clinical results with a new design. J Dent 39: 208–211. 21185905 10.1016/j.jdent.2010.12.005

[3] Sasse M, Kern M (2013) CAD/CAM single retainer zirconia-ceramic resin-bonded fixed dental prostheses: clinical outcome after 5 years. Int J Computerized Dent 16: 109–118. 23930573

[4] Thompson JY, Stoner BR, Piascik JR, Smith R (2011) Adhesion/cementation to zirconia and other non-silicate ceramics: where are we now? Dent Mater 27: 71–82. 21094526 10.1016/j.dental.2010.10.022 PMC3046396

[5] Piconi C, Maccauro G (1999) Zirconia as a ceramic biomaterial. Biomaterials 20: 1–25. 9916767 10.1016/s0142-9612(98)00010-6

[6] Piascik JR, Swift EJ, Thompson JY, Grego S, Stoner BR (2009) Surface modification for enhanced silanation of zirconia ceramics. Dent Mater 25: 1116–21. 19376572 10.1016/j.dental.2009.03.008 PMC2720441

[7] Papia E, Larsson C, du Toit M, Vult von Steyern P (2014) Bonding between oxide ceramics and adhesive cement systems: a systematic review. J Biomed Mater Res B Appl Biomater 102: 395–413. 24123837 10.1002/jbm.b.33013

[8] Piascik JR, Swift EJ, Braswell K, Stoner BR (2012) Surface fluorination of zirconia: adhesive bond strength comparison to commercial primers. Dent Mater 28: 604–8. 22364644 10.1016/j.dental.2012.01.008

[9] Spohr AM, Borges GA, Junior LH, Mota EG, Oshima HM (2008) Surface modification of In-Ceram Zirconia ceramic by Nd:YAG laser, Rocatec system, or aluminum oxide sandblasting and its bond strength to a resin cement. Photomed Laser Surg 26: 203–8. 18588435 10.1089/pho.2007.2130

[10] Aboushelib MN, Kleverlaan CJ, Feilzer AJ (2007) Selective infiltration-etching technique for a strong and durable bond of resin cements to zirconia-based materials. J Prosthet Dent 98: 379–88. 18021827 10.1016/S0022-3913(07)60123-1

[11] Atsu SS, Kilicarslan MA, Kucukesmen HC, Aka PS (2006) Effect of zirconium-oxide ceramic surface treatments on the bond strength to adhesive resin. J Prosthet Dent 95: 430–6. 16765155 10.1016/j.prosdent.2006.03.016

[12] Kumbuloglu O, Lassila LV, User A, Vallittu PK (2006) Bonding of resin composite luting cements to zirconium oxide by two air-particle abrasion methods. Oper Dent 31: 248–55. 16827029 10.2341/05-22

[13] Chen C, Kleverlaan CJ, Feilzer AJ (2012) Effect of an experimental zirconia-silica coating technique on micro tensile bond strength of zirconia in different priming conditions. Dent Mater 28: e127–34. 22578988 10.1016/j.dental.2012.04.020

[14] Kern M, Wegner SM (1998) Bonding to zirconia ceramic: adhesion methods and their durability. Dent Mater 14: 64–71. 9972153 10.1016/s0109-5641(98)00011-6

[15] Attia A, Kern M (2011) Long-term resin bonding to zirconia ceramic with a new universal primer. J Prosthet Dent 106: 319–27. 22024182 10.1016/S0022-3913(11)60137-6

[16] Kern M, Barloi A, Yang B (2009) Surface conditioning influences zirconia ceramic bonding. J Dent Res 88: 817–22. 19767578 10.1177/0022034509340881

[17] Tanaka R, Fujishima A, Shibata Y, Manabe A, Miyazaki T (2008) Cooperation of phosphate monomer and silica modification on zirconia. J Dent Res 87: 666–70. 18573988 10.1177/154405910808700705

[18] Aboushelib MN (2011) Evaluation of zirconia/resin bond strength and interface quality using a new technique. J Adhes Dent 13: 255–60. 21734959 10.3290/j.jad.a19241

[19] Chen L, Suh BI, Brown D, Chen X (2012) Bonding of primed zirconia ceramics: evidence of chemical bonding and improved bond strengths. Am J Dent 25: 103–8. 22779284

[20] Carriere D, Moreau M, Barboux P, Boilot JP, Spalla O (2004) Modification of the surface properties of porous nanometric zirconia particles by covalent grafting. Langmuir 20: 3449–55. 15875881 10.1021/la036249m

[21] Kitayama S, Nikaido T, Takahashi R, Zhu L, Ikeda M, et al (2010) Effect of primer treatment on bonding of resin cements to zirconia ceramic. Dent Mater 26: 426–32. 20102781 10.1016/j.dental.2009.11.159

[22] Chen L, Shen H, Suh BI (2013) Effect of incorporating BisGMA resin on the bonding properties of silane and zirconia primers. J Prosthet Dent 110: 402–7. 24007793 10.1016/j.prosdent.2013.04.005

[23] Felizardo KR, Lemos LV, de Carvalho RV, Gonini Junior A, Lopes MB, et al (2011) Bond strength of HEMA-containing versus HEMA-free self-etch adhesive systems to dentin. Braz Dent J 22: 468–72. 22189641 10.1590/s0103-64402011000600005

[24] Magne P, Paranhos MP, Burnett LH Jr (2010) New zirconia primer improves bond strength of resin-based cements. Dent Mater 26: 345–52. 20047757 10.1016/j.dental.2009.12.005

[25] Bail M, Malacarne-Zanon J, Silva SM, Anauate-Netto A, Nascimento FD, et al (2012) Effect of air-drying on the solvent evaporation, degree of conversion and water sorption/solubility of dental adhesive models. J Mater Sci Mater Med 23: 629–38. 22210310 10.1007/s10856-011-4541-y

[26] Queiroz JR, Benetti P, Ozcan M, de Oliveira LF, Della Bona A, et al (2012) Surface characterization of feldspathic ceramic using ATR FT-IR and ellipsometry after various silanization protocols. Dent Mater 28: 189–96. 22035984 10.1016/j.dental.2011.10.009

[27] Monticelli F, Toledano M, Osorio R, Ferrari M (2006) Effect of temperature on the silane coupling agents when bonding core resin to quartz fiber posts. Dent Mater 22: 1024–8. 16364422 10.1016/j.dental.2005.11.024

[28] Papacchini F, Monticelli F, Hasa I, Radovic I, Fabianelli A, et al (2007) Effect of air-drying temperature on the effectiveness of silane primers and coupling blends in the repair of a microhybrid resin composite. J Adhes Dent 9: 391–7. 17847642

[29] Ikeda T, De Munck J, Shirai K, Hikita K, Inoue S, et al (2008) Effect of air-drying and solvent evaporation on the strength of HEMA-rich versus HEMA-free one-step adhesives. Dent Mater 24: 1316–23. 18423839 10.1016/j.dental.2008.02.009

[30] Ozcan M, Matinlinna JP, Vallittu PK, Huysmans MC (2004) Effect of drying time of 3-methacryloxypropyltrimethoxysilane on the shear bond strength of a composite resin to silica-coated base/noble alloys. Dent Mater 20: 586–90. 15134947 10.1016/j.dental.2003.10.003

[31] Kim MJ, Kim YK, Kim KH, Kwon TY (2011) Shear bond strengths of various luting cements to zirconia ceramic: surface chemical aspects. J Dent 39: 795–803. 21907260 10.1016/j.jdent.2011.08.012

[32] Wolfart M, Lehmann F, Wolfart S, Kern M (2007) Durability of the resin bond strength to zirconia ceramic after using different surface conditioning methods. Dent Mater 23: 45–50. 16427692 10.1016/j.dental.2005.11.040

[33] Inokoshi M, Kameyama A, De Munck J, Minakuchi S, Van Meerbeek B (2013) Durable bonding to mechanically and/or chemically pre-treated dental zirconia. J Dent 41: 170–9. 23137995 10.1016/j.jdent.2012.10.017

[34] Gomes AL, Castillo-Oyague R, Lynch CD, Montero J, Albaladejo A (2013) Influence of sandblasting granulometry and resin cement composition on microtensile bond strength to zirconia ceramic for dental prosthetic frameworks. J Dent 41: 31–41. 23022106 10.1016/j.jdent.2012.09.013

[35] Yoshida K, Tsuo Y, Atsuta M (2006) Bonding of dual-cured resin cement to zirconia ceramic using phosphate acid ester monomer and zirconate coupler. J Biomed Mater Res B Appl Biomater 77: 28–33. 16193486 10.1002/jbm.b.30424

[36] Yang B, Barloi A, Kern M (2010) Influence of air-abrasion on zirconia ceramic bonding using an adhesive composite resin. Dent Mater 26: 44–50. 19766300 10.1016/j.dental.2009.08.008

[37] Zandparsa R, Talua NA, Finkelman MD, Schaus SE (2014) An In Vitro Comparison of Shear Bond Strength of Zirconia to Enamel Using Different Surface Treatments. J Prosthodont 23: 117–23. 23890275 10.1111/jopr.12075

[38] Perdigao J, Fernandes SD, Pinto AM, Oliveira FA (2013) Effect of artificial aging and surface treatment on bond strengths to dental zirconia. Oper Dent 38: 168–76. 22788723 10.2341/11-489-L

[39] Van Landuyt KL, De Munck J, Snauwaert J, Coutinho E, Poitevin A, et al (2005) Monomer-solvent phase separation in one-step self-etch adhesives. J Dent Res 84: 183–8. 15668338 10.1177/154405910508400214

[40] Yiu CK, Pashley EL, Hiraishi N, King NM, Goracci C, et al (2005) Solvent and water retention in dental adhesive blends after evaporation. Biomaterials 26: 6863–72. 15964621 10.1016/j.biomaterials.2005.05.011

[41] Hiraishi N, Breschi L, Prati C, Ferrari M, Tagami J, et al (2007) Technique sensitivity associated with air-drying of HEMA-free, single-bottle, one-step self-etch adhesives. Dent Mater 23: 498–505. 16690113 10.1016/j.dental.2006.03.007

[42] Luthy H, Loeffel O, Hammerle CH (2006) Effect of thermocycling on bond strength of luting cements to zirconia ceramic. Dent Mater 22: 195–200. 16143382 10.1016/j.dental.2005.04.016

