# Supplementary figures and images for: Bonding of Resin Cement to Zirconia with High Pressure Primer Coating
Source: PLoS One. 2014 Jul 3;9(7):e101174. doi: 10.1371/journal.pone.0101174 (PMC4081122; doi:10.1371/journal.pone.0101174)

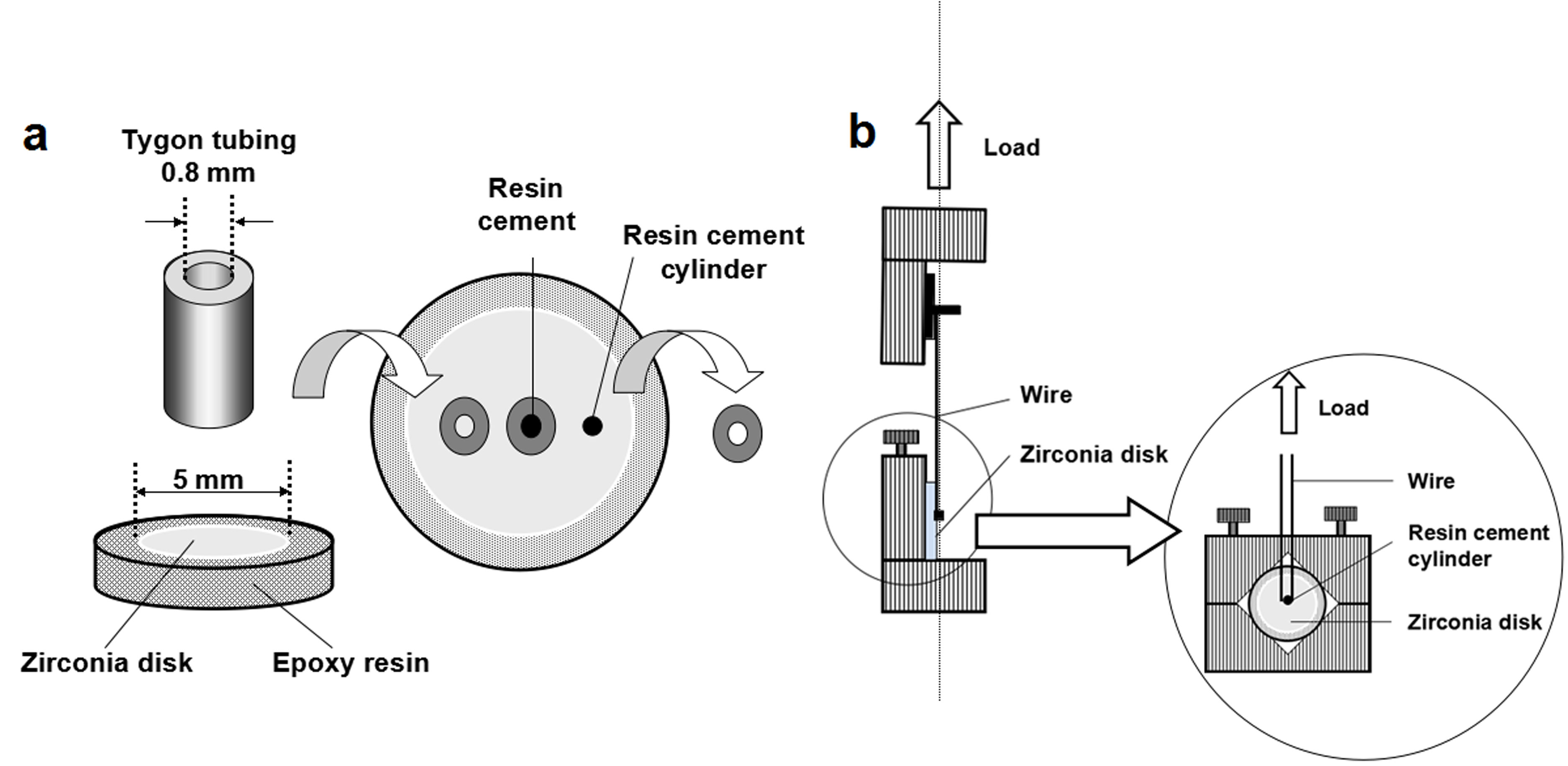

Supplement: Figure S1 — Schematic diagram of micro-shear test. (a) Prior to micro-shear bond test, the tygon tubes around the resin cement cylinders were removed, by gently cutting the tubes into two hemi-cylinders using a feather blade. (b) Specimens were clamped with the custom fixture and tested in a universal testing machine (EZ-Test 500 N, Shimadzu, Kyoto, Japan). A thin wire (diameter 0.2 mm) was looped around the resin cement cylinder, making contact through half of its circumference and was gently held flush against the resin/zirconia interface. A shear force was applied to each specimen at a cross-head speed of 0.5 mm/min until failure occurred. The resin-zirconia interface, the wire loop and the center of the load cell were aligned as straight as possible to ensure the desired orientation of the applied shear test force. (TIF) [file pone.0101174.s001.tif]
