# Supplementary material for: Bonding of Resin Cement to Zirconia with High Pressure Primer Coating
Source: PLoS One. 2014 Jul 3;9(7):e101174. doi: 10.1371/journal.pone.0101174 (PMC4081122; doi:10.1371/journal.pone.0101174)
Supplement: Table S1 — List of materials used in the present study. (DOC) [file pone.0101174.s002.doc]

**Table S1 List of materials used in the present study**

| Materials | Major composition^a^ | Batch number | Manufacturer |
| --- | --- | --- | --- |
| Katana Zirconia | Zirconium dioxide: 94.4 wt%  Yttrium trioxide: 5.6 wt% | 200218 | Noritake Dental Supply Co, Ltd, Miyoshi, Japan |
| Clearfil SA Luting | Paste A: BisGMA, TEGDMA, 10-MDP, DMA, silanated barium glass filler, silanated colloidal silica  Paste B: BisGMA, DMA, silanated barium glass filler, silanated colloidal silica, surface-treated sodium fluoride | 143BA | Kuraray Medical Inc., Tokyo, Japan |
| Clearfil Ceramic Primer | 3-MPS, 10-MDP, ethanol | 00006C | Kuraray Medical Inc. |
| Z-Prime Plus | 10-MDP, carboxylic acid resin monomer, BisGMA, HEMA, other resin monomers | REF 586-191-C | Bisco Inc., Schaumburg, IL, USA |

^a^Composition information provided by the manufacturers.

Abbreviations - 3-MPS: 3-methacryloxypropyltrimethoxysilane; BisGMA: bisphenol A diglycidyl methacrylate; DMA: aliphatic dimethacrylate; HEMA: 2-hydroxyethyl methacrylate; 10-MDP: 10-methacryloyloxydecyl dihydrogen phosphate; TEGDMA: triethylene glycol dimethacrylate
