# Supplementary material for: Bonding of Resin Cement to Zirconia with High Pressure Primer Coating
Source: PLoS One. 2014 Jul 3;9(7):e101174. doi: 10.1371/journal.pone.0101174 (PMC4081122; doi:10.1371/journal.pone.0101174)
Supplement: Table S2 — Subgroups and primer-coating protocols used in the present study. (DOC) [file pone.0101174.s003.doc]

**Table S2** Subgroups and primer-coating protocols used in the present study

| Primer-coating protocols | | Group designation |
| --- | --- | --- |
| Zirconia primer | Air-drying pressure |  |
| Clearfil Ceramic Primer | 0.1 MPa (gentle) | CCP-0.1 |
|  | 0.2 MPa (intermediate) | CCP-0.2 |
|  | 0.3 MPa (high) | CCP-0.3 |
|  | 0.4 MPa (high) | CCP-0.4 |
|  |  |  |
| Z-Prime Plus | 0.1 MPa (gentle) | ZPP-0.1 |
|  | 0.2 MPa (intermediate) | ZPP-0.2 |
|  | 0.3 MPa (high) | ZPP-0.3 |
|  | 0.4 MPa (high) | ZPP-0.4 |
